# Correction: FragGen: towards 3D geometry reliable fragment-based molecular generation

**DOI:** 10.1039/d4sc90252a

**Published:** 2024-12-19

**Authors:** Odin Zhang, Yufei Huang, Shicheng Chen, Mengyao Yu, Xujun Zhang, Haitao Lin, Yundian Zeng, Mingyang Wang, Zhenxing Wu, Huifeng Zhao, Zaixi Zhang, Chenqing Hua, Yu Kang, Sunliang Cui, Peichen Pan, Chang-Yu Hsieh, Tingjun Hou

**Affiliations:** a College of Pharmaceutical Sciences, Zhejiang University Hangzhou 310058 Zhejiang China slcui@zju.edu.cn panpeichen@zju.edu.cn kimhsieh@zju.edu.cn tingjunhou@zju.edu.cn; b Zhejiang University Hangzhou 310058 Zhejiang China; c Anhui Province Key Lab of Big Data Analysis and Application, University of Science and Technology of China Hefei Anhui China; d Montreal Institute for Learning Algorithms, McGill University Montreal QC Canada

## Abstract

Correction for ‘FragGen: towards 3D geometry reliable fragment-based molecular generation’ by Odin Zhang *et al.*, *Chem. Sci.*, 2024, **15**, 19452–19465, https://doi.org/10.1039/D4SC04620J.

In the original publication of this article, the name of one of the authors was misspelled. The spelling is as follows:

Incorrect: [Shichen Cheng]

Correct: [Shicheng Chen]

The Royal Society of Chemistry apologises for these errors and any consequent inconvenience to authors and readers.

